# A cell-to-patient machine learning transfer approach uncovers novel basal-like breast cancer prognostic markers amongst alternative splice variants

**DOI:** 10.1186/s12915-021-01002-7

**Published:** 2021-04-12

**Authors:** Jean-Philippe Villemin, Claudio Lorenzi, Marie-Sarah Cabrillac, Andrew Oldfield, William Ritchie, Reini F. Luco

**Affiliations:** Institut de Génétique Humaine (IGH-UMR9002), Centre National de la Recherche Scientifique (CNRS), University of Montpellier, Montpellier, France

**Keywords:** Alternative splicing, Breast Cancer, Survival, Basal-like, Epithelial-to-mesenchymal transition, Machine learning classification

## Abstract

**Background:**

Breast cancer is amongst the 10 first causes of death in women worldwide. Around 20% of patients are misdiagnosed leading to early metastasis, resistance to treatment and relapse. Many clinical and gene expression profiles have been successfully used to classify breast tumours into 5 major types with different prognosis and sensitivity to specific treatments. Unfortunately, these profiles have failed to subclassify breast tumours into more subtypes to improve diagnostics and survival rate. Alternative splicing is emerging as a new source of highly specific biomarkers to classify tumours in different grades. Taking advantage of extensive public transcriptomics datasets in breast cancer cell lines (CCLE) and breast cancer tumours (TCGA), we have addressed the capacity of alternative splice variants to subclassify highly aggressive breast cancers.

**Results:**

Transcriptomics analysis of alternative splicing events between luminal, basal A and basal B breast cancer cell lines identified a unique splicing signature for a subtype of tumours, the basal B, whose classification is not in use in the clinic yet. Basal B cell lines, in contrast with luminal and basal A, are highly metastatic and express epithelial-to-mesenchymal (EMT) markers, which are hallmarks of cell invasion and resistance to drugs. By developing a semi-supervised machine learning approach, we transferred the molecular knowledge gained from these cell lines into patients to subclassify basal-like triple negative tumours into basal A- and basal B-like categories. Changes in splicing of 25 alternative exons, intimately related to EMT and cell invasion such as ENAH, CD44 and CTNND1, were sufficient to identify the basal-like patients with the worst prognosis. Moreover, patients expressing this basal B-specific splicing signature also expressed newly identified biomarkers of metastasis-initiating cells, like CD36, supporting a more invasive phenotype for this basal B-like breast cancer subtype.

**Conclusions:**

Using a novel machine learning approach, we have identified an EMT-related splicing signature capable of subclassifying the most aggressive type of breast cancer, which are basal-like triple negative tumours. This proof-of-concept demonstrates that the biological knowledge acquired from cell lines can be transferred to patients data for further clinical investigation. More studies, particularly in 3D culture and organoids, will increase the accuracy of this transfer of knowledge, which will open new perspectives into the development of novel therapeutic strategies and the further identification of specific biomarkers for drug resistance and cancer relapse.

**Supplementary Information:**

The online version contains supplementary material available at 10.1186/s12915-021-01002-7.

## Background

Breast cancer is a heterogenous disease with multiple molecular drivers and disrupted regulatory pathways [1, 2]. The development of large-scale genomics and transcriptomics methods has increased the capacity to identify clinically-relevant tumour subtypes with distinct molecular signatures. These can be used for a better choice of treatment and/or prediction of potential metastasis which can improve survival outcome [3, 4]. However, patients are still facing a high percentage of misdiagnosis in which undetected early metastasis and/or inappropriate choice of treatment can lead to deadly complications with the use of unnecessary severe chemotherapies or the apparition of drug resistance and subsequent tumour relapse [5]. Currently, breast cancer is classified into five major categories (normal-like, luminal A, luminal B, Her2-positive and basal-like) based on expression of three receptors: oestrogen and progesterone hormonal receptors (ER and PR) and the epidermal growth factor receptor ERBB2 (Her2). Basal-like are the most aggressive, and difficult to treat, type of breast cancer tumour. They are usually negative for the three receptors, and thus called triple negative breast cancer (TNBC), which represents 10–20% of all breast cancers. These tumours are usually found in younger patients with a larger size and higher probability of lymph node infiltration and metastasis [2, 6]. Furthermore, the absence of all three receptors reduces the number of targeted therapeutic strategies to be used, leaving nonspecific chemotherapy as the standard treatment of choice, which soon leads to dose-limiting side-effects, resistance to treatment and finally clinical relapse in less than 5 years [6]. A better understanding of the molecular differences in between these tumour categories will improve the choice of treatment and detection of early metastasis, which will significantly impact patient’s outcome. There have been many attempts to identify novel therapeutic targets and/or prognostic biomarkers to better subclassify breast cancer tumours [7]. Over 170 independent breast cancer susceptibility genomic variants have been identified. Many of which have been associated with a specific tumour category, such as ER positiveness or Her2 amplification. However, no clear subcategories exist despite tumour heterogeneity and differences in clinical response to treatment and tumour relapse within the same category [8–10]. Interestingly, alternative splicing is an emerging source of new biomarkers and therapeutic targets in cancer [11–15].

The alternative processing of mRNA precursors enables one gene to produce multiple protein isoforms with different functions, increasing protein diversity and the capacity of a cell to adapt to new environments. An increasing number of splice variants, and their respective splicing regulators, have been shown to confer a selective advantage to tumour cells. For instance, the splicing regulators RBM5, 6 and 10 favour tumour cell proliferation and colony formation by regulating the alternative splicing of the membrane-bound protein NUMB [16]. Post-translational activation of the splicing factor SRSF1 (also known as ASF/SF2) confers resistance to apoptosis by inducing inclusion of the anti-apoptotic splice variant in a network of functionally related genes, such as *Bcl-X* and *Mcl1* [17]. Regulation of VEGF splicing is detrimental for stimulation of angiogenesis [18]. A change in the alternative splicing of the pyruvate kinase pre-mRNA can switch tumour cells metabolism to adapt to the increased proliferation [19, 20]. Finally, a list of well-known alternatively spliced variants related to cell adhesion (CTNND1, CD44) and cytoskeleton organisation (ENAH, FLNB) is responsible for the acquisition of migratory and invasive phenotypes necessary for distal metastasis [13, 21–24]. The existence of functionally relevant cancer-specific isoforms is therefore a promising new source of highly specific and less toxic therapeutic targets for the development of isoform-specific antibodies and/or splice-switching antisense oligonucleotides [25, 26].

By taking advantage of an extensive transcriptomics and anti-tumour compound screening information publicly available in cancer cell lines from the Cancer Cell Line Encyclopedia (CCLE) [27], we identified a splicing signature that can stratify basal breast cancer cell lines into two well-known subtypes, basal A and basal B. In contrast to basal-like breast cancer patients, basal breast cancer cell lines are divided into two subgroups, basal A and basal B, depending on the expression profile of a subset of basal (cytokeratins, integrins), stem cell (CD44, CD24) and mesenchymal markers (Vimentin, fibronectin, MSN, TGFBR2, collagens, proteases) [28–30]. Basal B cell lines are mostly triple negative breast cancer cells that express classical mesenchymal and stem cell markers characteristic of the epithelial-to-mesenchymal transition (EMT), a biological process in which epithelial cells acquire mesenchymal features that are advantageous for the cancer cell, such as increased cell motility to invade distal organs in metastasis, resistance to apoptosis, refractory responses to chemotherapy and immunotherapy, and acquisition of stem cell-like properties like in cancer stem cells [31, 32]. In concordance, basal B cells are morphologically less differentiated, with a mesenchymal-like shape, and a more invasive phenotype in culture assays than basal A and luminal cells [28, 33, 34]. We aimed to transfer this basal A/basal B splicing classification into the clinic by using a semi-supervised machine learning approach. We successfully classified 40% of basal-like breast cancer patients (75/188) from the Cancer Genome Atlas (TCGA) [35] as basal B-like based on a unique 25 spliced gene signature characteristic of cells undergoing EMT. In this signature, we found well-known markers of malignancy, such as ENAH EMT splice variant that promotes lung metastasis [36] or CSF1 variant which promotes macrophage infiltration and distal metastasis [37], together with new promising splicing candidates of tumour progression and invasiveness (PLOD2, CTNND1, SPAG9). Finally, expression of this basal B signature was sufficient to identify triple negative breast cancer tumours with poor survival, highlighting the prognostic value of the newly identified splicing biomarkers to subclassify one of the most heterogenous and difficult to treat type of breast cancer. More studies in cell lines, particularly regarding resistance to treatment and cell invasion will be essential to refine this splicing signature in view of orienting treatment or predicting metastasis sites.

In conclusion, by adapting a machine learning approach, we were able to transfer the molecular knowledge obtained in experimental cell lines to identify novel biomarkers of poor prognosis and metastasis amongst triple negative breast cancers in patients. Furthermore, the study of the regulatory pathway involved in this specific splicing signature pointed to RBM47 as one of the splicing regulators responsible for the basal B-specific splicing signature, and for which differential expression levels also correlate with distinct prognostic values, turning this splicing factor a promising novel therapeutic target. Further clinical and functional validation of the 25 splicing events proposed in our basal B-specific splicing signature will open new perspectives in the understanding of triple negative breast cancers and the improvement of currently available therapeutic strategies and survival outcome.

## Results

### A distinctive basal B-like breast cancer splicing signature

**Fig. 1:**
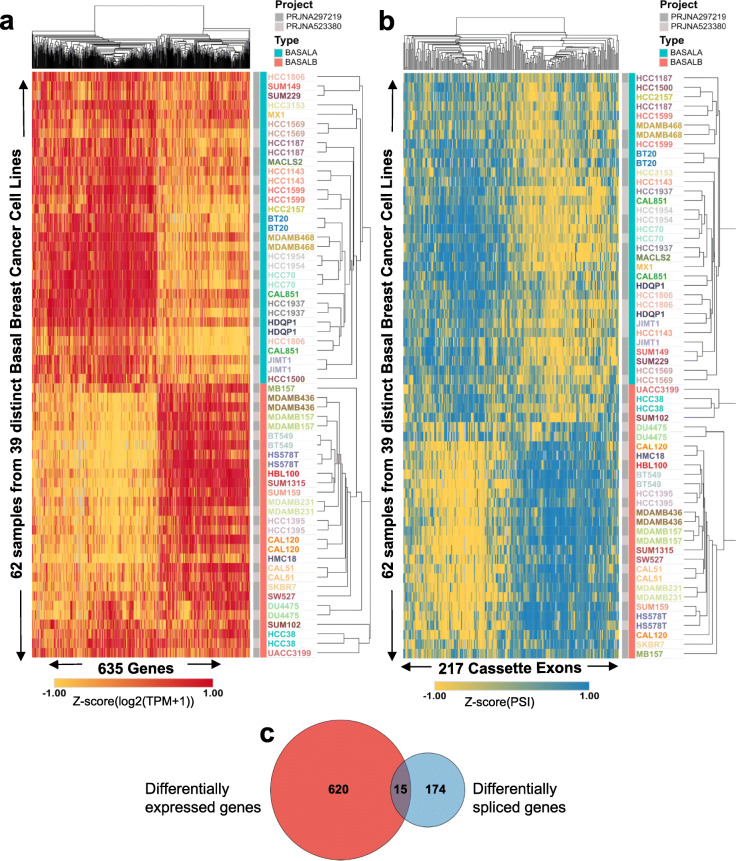
Basal cell lines are divided in two subgroups based on gene expression and splicing patterns. **a** Heatmap of the transcripts per million (TPM) values of the 635 genes which differential expression can cluster breast cancer cell lines into basal A and basal B (*P* value < 10^− 3^ by Kruskal-Wallis test). **b** Heatmap of the percentage spliced-in (PSI) values of the 217 exons which differential splicing can cluster breast cancer cell lines into basal A and basal B (*P* value < 10^− 3^ by Kruskal-Wallis test). **c** Venn Diagram of the genes differentially expressed and/or spliced between basal A and basal B cancer cell lines. The overlap is not higher than expected by Fisher’s exact test, two tail (*p* = 0.098)

**Fig. 2:**
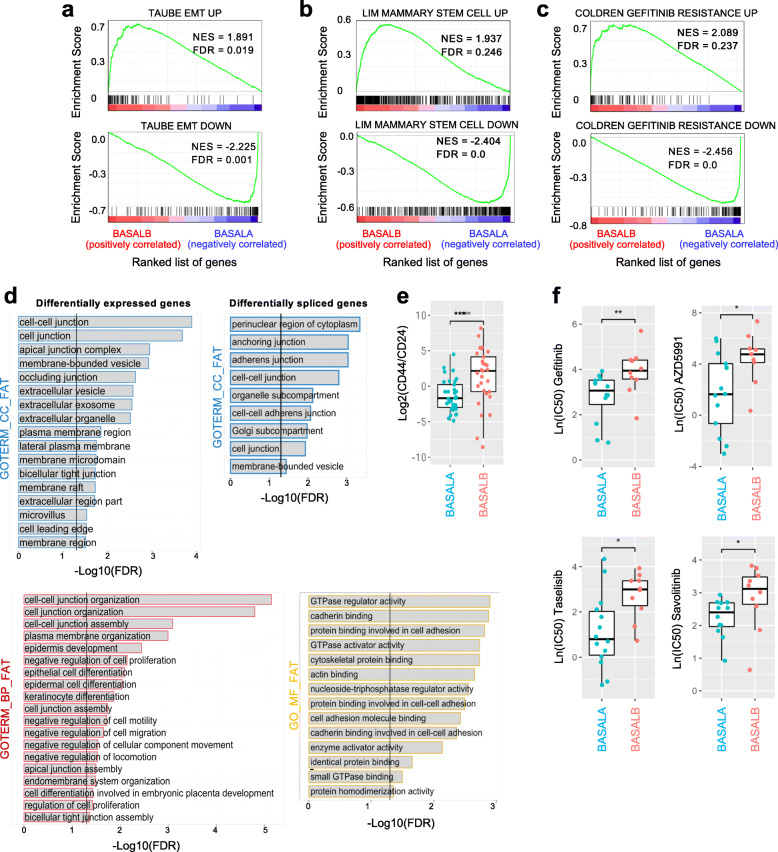
Basal B cell lines show mesenchymal, stem-like and resistance to treatment characteristics. **a**–**c** Gene Set Enrichment Analysis (GSEA) of differentially expressed genes between basal A and B cell lines for three different signatures: Mammary Stem Cell, EMT and Resistance to Gefitinib. Up-regulated genes in all signatures are enriched in basal B cell lines (FDR < 0.25). **d** Gene ontology analysis bar graphs for differentially expressed (left) and differentially spliced (right) genes between basal A and B cell lines. Gene ontology terms related to Cellular Component (GO_CC_FAT), Molecular Function (GO_MF_FAT) and Biological Process (GO_BP_FAT) are shown in the y axis in blue, yellow and red, respectively. Benjamini false discovery rate (FDR, −log10) is shown on the *x*-axis. Vertical lines mark an FDR threshold of FDR = 0.05 (−log10(0.05) = 1.3) for differentially expressed and spliced genes, respectively. **e.** Box plots of the median and 25th percentile of the CD44/CD24 log2 expression ratio for basal A and B cell lines. *P* value is calculated using the Wilcoxon rank-sum test. **f** Boxplots comparing IC50 values in basal A and B cell lines upon treatment with different drugs from the Genomics of Drug Sensitivity in Cancer 2 (GDS2) dataset. *P* values are calculated using the Wilcoxon rank-sum test

Data mining of large-scale genomics and transcriptomics datasets in breast cancer cell lines are a promising source of novel biomarker and therapeutic targets [23, 38, 39]. We sought to leverage the wealth of transcriptomics and functional data available in cancer cell lines to better understand different profiles of breast cancer. Hierarchical clustering of changes in alternative splicing of cassette exons and gene expression profile of 80 breast cancer cell lines from two extensive and complementary projects (Additional file 2: Table S1) revealed basal B cell lines as a distinctive group of cells with an expression and splicing profile significantly different from basal A and luminal cancer cells (Additional file 1: Fig. S1). To identify the transcriptional signature characteristic of basal B cells, we repeated the hierarchical clustering in just basal A and basal B cell lines to merge all the differentially expressed and spliced transcripts responsible for the segregation of basal B cell lines (Fig. 1). We found 635 genes and 217 spliced isoforms with significantly different levels between basal A and basal B cells (Fig. 1a, b). In line with published tissue-specific and EMT transcriptomics analyses [40–42], most of the genes differentially spliced were not affected at the expression level, suggesting that two different subsets of genes, and thus regulatory layers, are responsible for the basal B phenotype (Fig. 1c). Gene set enrichment analysis (GSEA) [43] between basal B and basal A cells confirmed the EMT and stem cell-like phenotype characteristic of basal B cell lines (Fig. 2a, b), which was supported with a higher CD44+/CD24− stem cell score (Fig. 2e) [28–30]. DAVID gene ontology analysis of differentially expressed and spliced genes also underlined biological terms that are hallmarks of EMT and cell invasiveness, such as cell-cell junction (Fig. 2d) [44]. However differentially expressed genes were also enriched in their own unique terms, related to extracellular vesicles/plasma membrane organisation. Whilst differentially spliced genes were specifically enriched in terms related to GTPase activity, cytoskeletal protein and cadherin binding, which reinforces the existence of two complementary regulatory pathways (Fig. 2d). Finally, another malignant characteristic acquired by cancer cells undergoing EMT is resistance to chemotherapy, which often leads to clinical relapse. Gene set enrichment analysis found upregulation of genes resistant to the Epidermal Growth Factor Receptor (EGFR) inhibitor Gefitinib (Fig. 2c), which is an alternative to hormonal therapy in Her2+ breast cancer tumours, but is not efficient in triple negative tumours [45]. Available drug assays from the Genome Drug Sensitivity in Cancer portal (GDSC) [46] confirmed the need of a higher concentration (IC50) of Gefitinib, and other EGFR inhibitors (Erlotinib, Sapitinib), to have the same deleterious effect on basal B compared to basal A cancer cells (Fig. 2f). Basal B cell lines also showed a significant resistance to well-known inhibitors of the cell cycle (irinotecan, taselisib, 5-fluorouracil), drug inducers of cell death (AZD5582, AZD5991) and other receptor tyrosine kinase inhibitors, such as savolitinib which inhibits c-MET to reduce tumour persistence and metastasis [47].

In summary, we have identified two distinct transcriptional and splicing signatures, specific of basal B cell lines, that underline an EMT phenotype with molecular characteristics related to cell invasion, stemness and resistance to chemotherapy. We next sought to investigate whether this basal B-specific splicing signature could also be used to subclassify basal-like/triple negative breast cancer patients.

### A semi-supervised machine learning approach to subclassify basal-like breast cancer patients

As a first and simple approach, we performed a hierarchical clustering followed by a k-means clustering (k = 2 for “A-like” and “B-like”) of the 188 patients, annotated as basal-like in The Cancer Genome Atlas Program (TCGA), using the 635 differentially expressed or 217 differentially spliced cassette exons characteristic of basal B cell lines (Additional file 1: Fig. S2a,b). Using such method, patients were forced to classify in one of the two groups based on differences in gene expression or splicing patterns. Since basal B cell lines show more invasive, cancer stem cell-like phenotypes, we assessed whether these aggressive characteristics were translated to the “B-like” patient group through differences in disease specific survival (DSS) rates. Kaplan-Meier analysis of DSS did not show significant differences between the two subgroups of basal-like patients (Additional file 1: Fig. S2c,d). However, we did observe a tendency for “B-like” patients to have a poor survival compared to “A-like” when just looking at differences in splicing, contrary to expression levels (*p* value = 0.09 vs 0.57, respectively—Additional file 1: Fig. S2c,d).

**Fig. 3:**
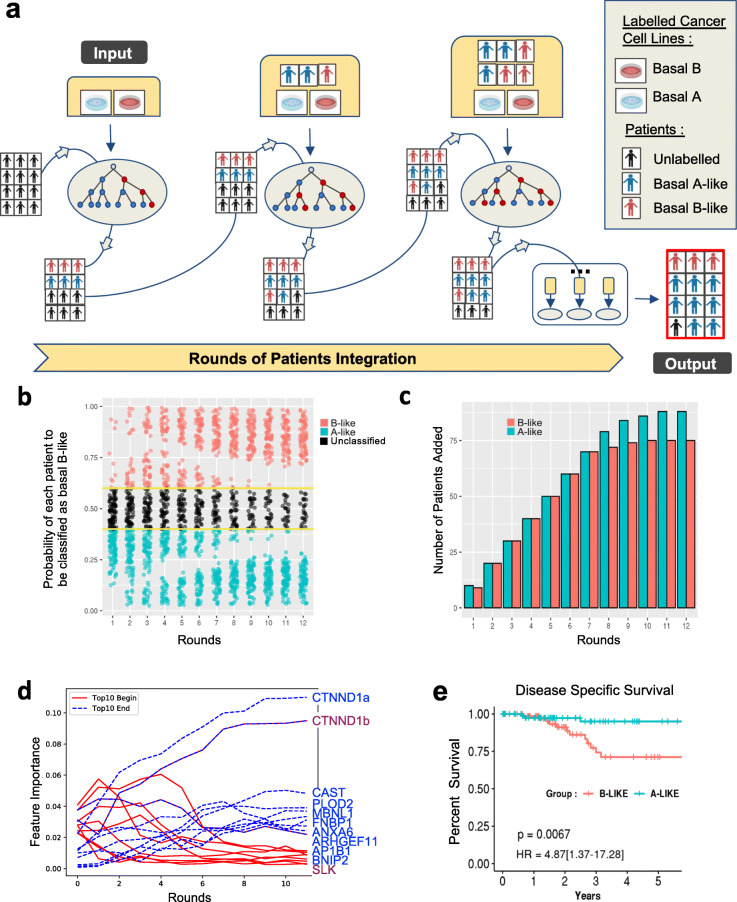
A Random Forest Classifier using knowledge transfer from cell lines to patients. **a.** Workflow scheme: a random forest (RF) model is built using cell lines labelled as basal B (red) or basal A (blue). It is then run iteratively, integrating at each round patients whose probability to be classified in one group or the other is amongst the ten highest. The classifier stops when no more patients can be classified. **b** Probability of a basal-like patient to be classified as basal B-like, basal A-like or unclassified over each round. Yellow lines indicate thresholds used to classify a patient as basal B-like (> 0.6) or basal A-like (< 0.4). **c** Bar plot of the number of patients added at each round. Patients with the highest probability to be classified are sequentially incorporated to the input cell lines in order to create a new classifier for the next round of integration. **d** Evolution of the feature importance at each round of iterative training. In red are the 10 splicing variants (features) most informative at the beginning of the transfer learning process. In blue are the 10 splicing variants most informative at the end. Only two exons remained informative from the beginning to the end (in blue and red). The name of the top 10 final most informative spliced genes are written in blue and in sequential order. **e** Kaplan-Meier plots of disease specific survival in basal A-like (blue) and basal B-like patients (red). Hazard ratio (HR) and logrank *p* value (*P*) discriminating the two groups are shown

In fact, it was not surprising that the transcript-level and splicing signatures did not translate directly from simplistic cell culture models to much more complex tumour patients with specific cell micro-environments and differences in cell heterogeneity. However, because the patients showed clear “A-like” and “B-like” signatures, we sought to develop a machine learning approach that would allow us to transfer part of the molecular and phenotypic observations found in cell-lines to patient data. Transfer learning is a recent research methodology that focuses on storing the knowledge gained when solving a problem, to apply it to a different, but related, one. Because we wanted to ensure that the newly developed cell-to-patient transfer learning algorithm could create interpretable models, we used a decision tree-based approach called Random Forest. In this cell-to-patient random forest classification method, we started by classifying basal A or basal B cell-lines based on their splicing and/or expression profile (Fig. 3a and Additional file 1: Figs. S3-S4). Then, once the model was trained on cell-lines, we would start integrating patient data gradually into the model. This was done iteratively by integrating at each round of classification the patients best predicted to be basal A-like and basal B-like, so their added informative value could be used back to train the system and improve the next round of classification (Fig. 3a). With this semi-supervised approach, the probability of assigning a patient to a specific subgroup evolves and improves at each round based on the updated information obtained from the best predicted patients, reaching at the end a stable population with the labels ‘basal A-like’, ‘basal B-like’ or ‘unclassified’ determined by the algorithm after 10–12 rounds (Fig. 3b,c and Additional file 1: Figs. S3b,c-S4b,c). Thanks to the gradual addition of patients at each round of training, there is a progressive increase, or decrease, in the feature importance of the splicing variants used to classify patients (Fig. 3d and Additional file 1: Figs. S3d-S4d). Out of the 188 basal-like patients, 75 were classified as basal B-like, 88 as basal A-like and 25 could not be classified based on their splicing signature. Using only expression levels, there was a slight biased towards the basal A-like phenotype, with 56 patients classified as basal B-like, 122 as basal A-like and 10 unclassified (Additional file 1: Fig. S3b-c). Combining differentially spliced and expressed features seemed to be the most performant classifier with 84 patients as basal B-like, 100 as basal A-like and just 4 unclassified (Additional file 1: Fig. S4b-c). Taken together, depending on the features used (splicing patterns, expression levels or both), patients were differently classified in basal A-like or basal B-like.

### An EMT-related basal B-specific splicing signature that marks poor prognosis

To address which classifier translates the best to patients the invasive, EMT-like and drug-resistant basal B phenotype found in cancer cells, we calculated the 5-year survival rate for each group of basal A-like and basal B-like issued from the three types of classification. Only basal B-like patients classified based on splicing levels had a poor prognosis compared to basal A-like patients (log-rank test *p* = 0.0067, HR = 4.87; 95% IC: [1.37–17.28] in Kaplan-Meier analysis and univariate Cox regression) (Fig. 3e). Basal B-like patients subclassified based on gene expression levels, or gene expression and splicing features, did not show significant differences in disease survival rate (Additional file 1: Fig.S3e-4e), suggesting that splicing biomarkers might be more informative to further subclassify basal-like patients based on prognosis. We thus decided to focus on the role of alternative splicing in identify triple negative basal-like breast cancer with poor prognosis.

**Fig. 4:**
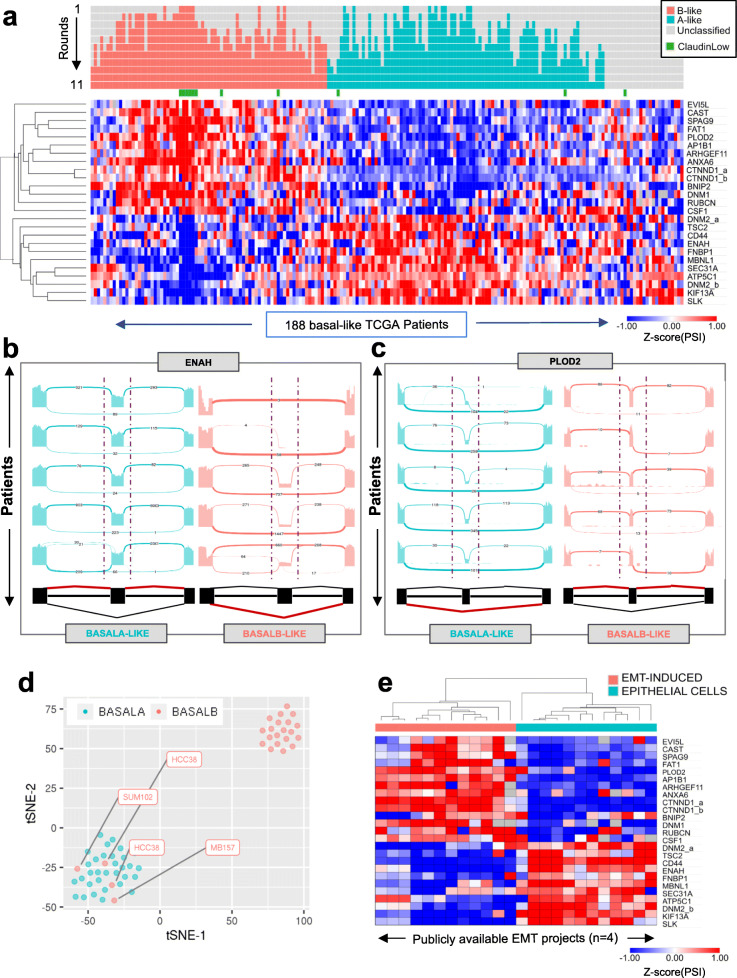
The basal B-specific splicing signature is associated to EMT features. **a** Heatmap of the Percentage Spliced-In (PSI) values of the 25 cassette exons most informative to classify TCGA basal-like patients into basal B-like (red) or basal A-like (blue). Claudin low tumours are highlighted in green. **b**, **c** Sashimi plots displaying ENAH and PLOD2 splicing patterns in randomly selected patients classified as basal A-like and basal B-like. **d** Changes in alternative splicing of these 25 basal B-specific splicing events is sufficient to properly cluster 55 basal breast cancer cell lines from 3 unrelated sequencing projects into basal B and basal A using t-SNE. Of note, three basal B cell lines, HCC38, MDA-MB-157 and SUM102 were misclassified as Basal A cell lines (red dots). Although HCC38 has also been classified as Basal A in the DepMap portal (www.depmap.org). **e** Heatmap of the PSI values of the 25 basal B-specific splicing signature in public RNA-seq datasets from four different EMT projects. Basal B-like events have the same splicing patterns as EMT-induced cells

To extract the most informative splicing features from the cell-to-patient transfer learning classifier, we used the Boruta feature selection method [48]. This allowed us to select the key splicing events responsible for the basal A/B classification without the need to predefine arbitrary thresholds (Fig. 4a). Out of the 217 differentially spliced exons between basal A/B cell lines, just 25 were needed to subclassify breast cancer patients in basal A or basal B-like tumours (Fig. 4a and Additional file 3: Table S2). Sashimi plots representing the splicing patterns of some of these basal B-specific splicing events, such as the well-known splicing biomarker of cancer metastasis ENAH [26] and the newly identified splicing biomarkers PLOD2, SPAG9 and KIF13a, validated the observed changes in splicing between basal A and basal B-like patients (Fig. 4b-c and Additional file 1: Fig. S5a-b). Moreover, the changes in percentage of spliced-in (PSI) of the 25 basal B-specific splicing events between the two subtypes of basal-like patients correlated with the observed splicing changes between basal A/B cell lines (Additional file 1: Fig. S5c-d), further supporting the transfer of knowledge from the laboratory to the clinic. Finally, in the absence of publicly available RNA-seq data on a second cohort of basal-like breast cancer patients, we took advantage of three independent sequencing projects on breast cancer cell lines, different from the ones used for the training of the semi-supervised classifier (Additional file 2: Table S1). Distribution of 52 independent breast cancer cell lines showed a 93% accuracy in the spatial segregation (t-SNE) of basal A from basal B cells based on the splicing pattern of the 25 newly identified splicing events (Fig. 4d). Just three cell lines were misclassified as basal A (HCC38, SUM102 and MDA-MB-157). It is worth noting that one of these, HCC38, was also labelled as basal A in the DepMap portal (www.depmap.org), which validated our methodology and the specificity of the splicing signature towards a basal B-like phenotype.

**Fig. 5:**
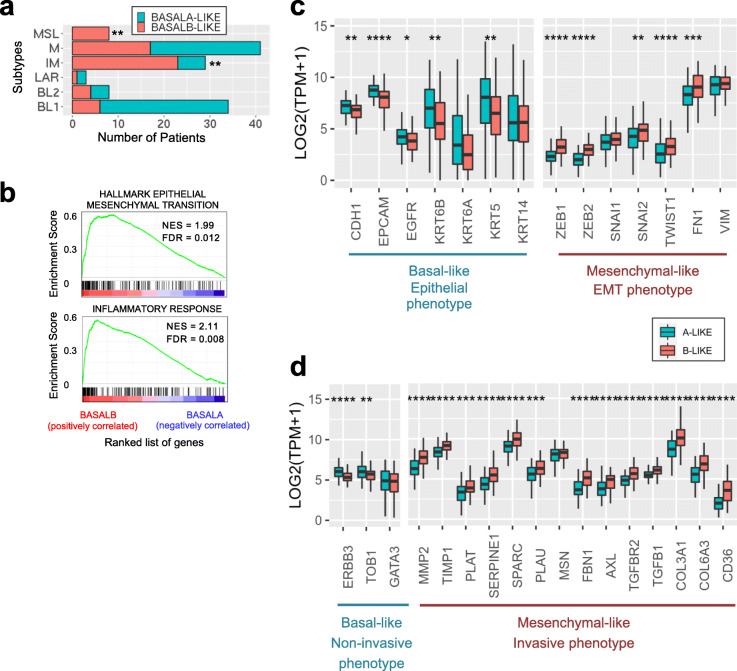
Basal B-like patients express hallmarks of EMT and metastasis that leads to a poor prognosis. **a** Lehman classification for basal A- and B-like patients. **p < 0.01 in Fisher’s exact test, two tail, comparing basal B to basal A. **b** Gene Set Enrichment Analysis (GSEA) of the genes differentially expressed between basal A- and B-like patients. Hallmark EMT and inflammatory response signatures are enriched in basal B-like patients. **c.** Box plots of the median and 25th percentile of the expression levels (in TPM) of major epithelial and mesenchymal-like EMT markers in basal A-like (blue) and basal B-like (red) patients. **d** Box plot of the mean and 25th percentile of the expression levels (in TPM) of Basal-like non-invasive and mesenchymal-like invasive markers in basal A-like (blue) and basal B-like (red) patients. ** *P* < 0.01, *** *P* < 0.001, **** *P* < 0.0001 in Wilcoxon rank-sum test comparing basal A-like to basal B-like

Consistent with basal B cell lines being more mesenchymal, differences in the alternative splicing of these 25 basal B-specific splicing events in four different cellular models of EMT, coming from different cell types and methods of EMT induction [49–52], successfully clustered epithelial cells from mesenchymal with a pattern of splicing equivalent to basal A and basal B-like patients, respectively (Fig. 4e). Of note, another 25 gene-based EMT-like splicing signature characteristic of luminal breast cancer tumours has also been identified capable of subclassifying mesenchymal-like breast cancer tumours with poor prognosis [38]. Consistent with a more luminal-specific signature, despite both marking EMT phenotypes, not more than six splicing events were found in common between the two splicing signatures (ATP5C1, CTNND1, KIF13a, PLOD2, SEC31a and SPAG9), which further supports the specificity of our newly identified splicing signature for basal-like triple negative breast cancer. Finally, using one of the first established molecular subtypes of triple negative breast cancer tumours based on gene expression, which is the Lehman classification [53], we found that basal B-like patients are mostly found in the categories associated with mesenchymal stem-like (MSL) and immunomodulatory (IM) subtypes (Fig. 5a), which goes in line with a gene set enrichment of terms related to inflammatory responses and hallmark of EMT (Fig. 5b).

When looking at the expression of well-known basal and EMT biomarkers in the two subpopulations of basal A/B-like patients, we found that basal A-like patients express classical basal/epithelial markers, such as E-cadherin, EPCAM and cytokeratin KRT5/KRT6/KRT14, together with ERBB3 and TOB1 which are markers of more differentiated, non-invasive cells [2]. On the other hand, basal B-like patients express classical EMT/mesenchymal markers such as Fibronectin, the EMT inducers Twist and Slug, and the Zinc-finger transcriptional regulators Zeb1 and Zeb2 which have recently been shown to confer stemness properties that can increase the plasticity and invasive capacity of the tumour cells [54] (Fig. 5c, d). In line with a more aggressive, invasive phenotype, basal B-like patients express cytoskeletal (MSN, FN1) and extracellular matrix signalling proteins (TGFB1, TGFBR2, FBN1, AXL), collagens (COL3A1, COL6A3) and proteases (MMP2, TIMP1, CTSC, PLAU, SERPINE1/2, PLAT), which are necessary for cell’s migration and dissemination to distal organs during metastasis [2]. Finally, basal B-like patients overexpress a recently identified new marker of metastasis-initiating cells, the fatty acid receptor CD36 [20]. Clinically, the presence of CD36-positive cells has been correlated with a lower survival rate in many carcinomas, including breast cancer, and inhibition of CD36 impairs metastasis in breast cancer-derived tumours, turning this receptor into an important biomarker of tumour cell dissemination and a potential new target to reduce cell invasion. The fact that basal B-like tumour cells co-express this metastasis-initiating marker further strengthens the aggressive nature of this tumour subclass and the clinical relevance of the basal B-specific splicing signature in tumour progression and relapse.

Overall, we have identified a novel splicing signature, specific of triple negative breast cancer tumours, that marks patients with the poorest prognosis. This basal B-like splicing signature is responsible of a stem-like, EMT phenotype that favours tumour growth, invasion of distal organs and increased drug resistance, which eventually leads to tumour relapse and metastasis. Interestingly, some of the genes differentially expressed in these basal B-like patients are well-known markers of metastasis-initiating cells, such as the alternatively spliced CTNND1 and PLOD2 genes or the fatty acid receptor CD36, turning these biomarkers into promising new targets for innovative therapies, such as the use of splicing specific antibodies [6, 26].

### A metastasis-related common regulatory pathway for the basal B-specific splicing signature

**Fig. 6:**
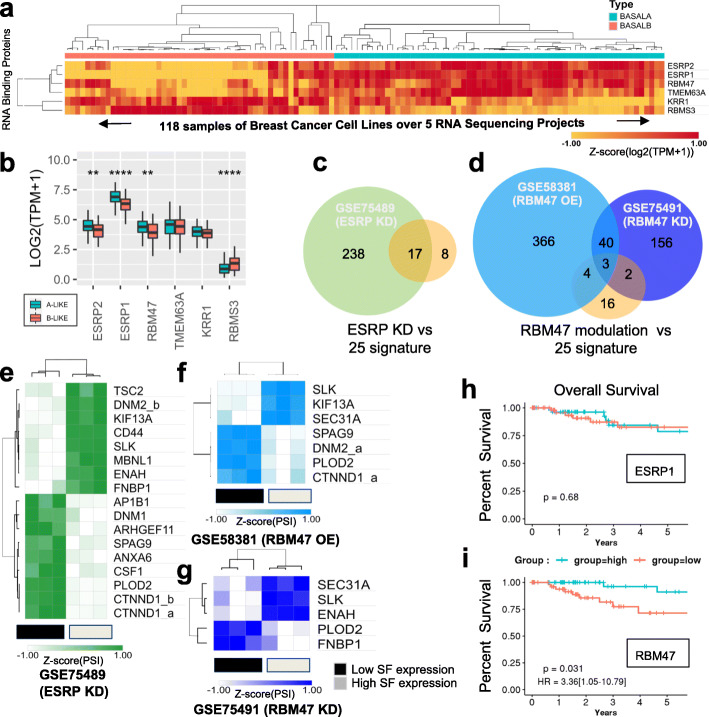
The basal B-specific splicing signature is co-regulated by ESRP1 and RBM47. **a** Heatmap of transcripts per million values for RNA binding proteins (RBP) differentially expressed in basal A and basal B cell lines (*P* value < 10^− 9^ by Kruskal-Wallis test). **b** Box plots of the mean and 25th percentile of the expression levels (in TPM) of the same RBP as in a, but in basal A-like and basal B-like patients. **c**, **d** Venn diagrams of the number of splicing events from the basal B-specific splicing signature dependent on the splicing factors (SF) ESRP1/2 and RBM47 using a cutoff of |DeltaPsi| > 0.1 and a higher probability > = 0.95. **e**–**g** Heatmaps of the PSI values of the ESRP and RBM47-dependent exons from c and d in ESRP1/2 knock downed H358 cells, RBM47 overexpressed MDA-MB-231 cells and RBM47 knock downed H358 cells. **h**, **i** Kaplan-Meier plots for overall survival in basal-like TCGA patients expressing the highest tercile (blue) or the lowest tercile (red) of ESRP1 and RBM47 expression levels. HR (hazard ratio) and logrank *p* values (P) discriminating between groups are shown

Hierarchical clustering of basal A and B cell lines based on the differential expression of RNA-binding proteins highlighted six RNA regulators, ESRP1, ESRP2, RBM47, TMEM63A, KRR1 and RBMS3 (Fig. 6a) (Kruskal-Wallis *p* < 10^−9^). Interestingly, ESRP1/2 and RBM47 are significantly less expressed in basal B-like than basal A-like patients (Fig. 6b), consistently with the known inhibitory effect of these three splicing regulators in EMT progression and metastasis [52, 55, 56]. Available transcriptomics data in ESRP1/2 and RBM47 lung carcinoma NCI-H358-depleted cells [52] and RBM47 overexpressing breast cancer metastatic MDA-MB-231 cells [57] showed that 19 of the 25 splicing events responsible for the newly identified basal B-specific splicing signature could potentially be regulated by ESRP1/2 and/or RBM47 in breast cancer cells (Fig. 6c, d). Importantly, in the cell types analysed, ESRP1/2 and RBM47 induce the epithelial, basal A-like splicing phenotype, suggesting a potential tumour suppressor effect for these splicing regulators (Figs. 6e–g, 4e and Additional file 1: S5c-d). Consistently with this observation, low expression of RBM47 in basal-like breast cancer patients was associated with poor overall survival (log-rank test *p* = 0.031, HR = 3.36, 95% IC:[1.05–10.79] Fig. 6h, i), which supports previous experimental evidence of a role for RBM47 in supressing breast cancer metastasis and progression [56]. In fact, RBM47-dependent basal B-specific splicing events were found to be functionally interconnected by physical and/or genetic interactions, which points to the existence of a common basal B-specific regulatory network associated with tumour malignancy (Additional file 1: Fig. S6a). In support, most of RBM47-dependent basal B-specific splicing events play well-known roles in cell-cell adhesion (CTNND1) [58], cytoskeleton organisation (ENAH, SLK, FNBP1) [59, 60], endocytosis (KIF13A, DNM2) [61] and association with the extracellular matrix (PLOD2) [62], which are all key processes for gaining the cell motility and invasiveness necessary in tumour metastasis (54–58). Of note, expression of just one of these basal B-specific splice variants, which are CTNND1, ENAH and PLOD2, is sufficient to lower the disease-specific survival rate of basal B-like breast cancer patients compared to basal A-like (Additional file 1: Fig. S6b-g). These splicing events could turn into promising new therapeutic strategies aiming at specific key regulatory genes instead of a pleiotropic splicing regulator that could have unsuspected secondary effects.

In summary, by taking advantage of extensive large-scale transcriptomics data from breast cancer cell lines and patients, we identified the first splicing signature capable of subclassifying basal-like tumours based on their aggressiveness and drug resistance. Importantly, novel splicing biomarkers of poor prognosis were identified that should be further studied in more functional assays to test their capacity to inhibit tumour invasion and metastasis. Results from these assays will open new perspectives in the development of improved target therapies and more accurate diagnostic profiles to identify the basal-like triple negative breast cancer patients with a higher chance of relapse.

## Discussion

Cancer-specific dysregulation of alternative splicing is a promising source of cancer biomarkers and therapeutic targets to improve diagnostics and thus overall survival rate [63]. An increasing number of mutations at core spliceosome components, such as S3FB1 and U2AF1, or upregulation of specific splicing factors, such as SRSF1 and other members of the SR protein family, which are now considered oncogenes, have been intimately linked to tumour progression and malignancy [64]. Furthermore, an increasing number of alternatively spliced events, like CD44, ENAH, CTNND1 and FLNB, have been shown to impact cell invasion and metastasis on their own, making them promising new targets for more specific therapeutic strategies compared to the inhibition of splicing regulators [22, 23, 65, 66]. Effectively, splicing regulators are not only responsible for the regulation of splicing of a subset of genes, but they are also responsible for other RNA related functions such as translation, mRNA export and nonsense-mediated mRNA decay [56, 64], which can have numerous downstream deleterious effects when inhibited in a targeted therapy. By specifically targeting a key downstream splicing event, as in splicing-specific immunotherapy, a more cancer-specific and direct impact on the cell phenotype might be achieved (134, 135).

Large scale public molecular data sets on genomics (copy number and mutation), epigenomics, transcriptomics, proteomics, in vitro and in vivo cell invasiveness and response to anti-tumour compounds in a large number of patients (11,000 patients across 33 different tumour types from the Genome Cancer Atlas) and human-derived cell lines (1000 cancer cell lines across 36 tumour types from the Broad Institute’s Cancer Cell Line Encyclopedia) has become an extraordinary toolbox to identify novel prognostic markers of early metastasis and/or resistance to specific drugs, which are the two major reasons for clinical relapse and low survival rate [67–69]. Unfortunately, the translatability of these pre-clinical findings is often limited since culture cells are not representative of the variety of individuals nor the biological reality of the tumour’s multicellular environment. Yet, culture procedures are improving with the creation of organoids, and machine learning approaches combined with large-scale data mining are bypassing some of these important caveats. This is the case of our cell-to-patient random forest classifier approach, in which the addition at each round of selection of novel informative features, based on the patients classified in previous rounds, allows an algorithm to make use of the information learned from cell lines. Thanks to this approach, we were able to identify the first splicing signature, composed of 25 alternatively spliced exons, capable of subclassifying basal-like breast cancer patients into two subtypes with different prognoses: basal A- and basal B-like.

Actually, this newly identified basal B-like splicing signature underlined a stem cell-like EMT signature, with hallmarks of cell invasiveness and drug resistance. Five of these 25 alternatively spliced genes are well-known to play a role in cancer (ARHGEF11, CD44, CTNND1, ENAH, MBNL1) [70–72]. Six have been indirectly linked to tumour malignancy and are thus new splicing targets to study (CAST, CSF1, PLOD2, SLK, SPAG9, TSC2) [60, 62, 73–76]. The rest are completely unknown for their splicing role in cancer, even though changes in expression of some of them have been shown to play a role in tumour progression, chemosensitivity and metastasis without specifically addressing which splice variant (ATP5C1, BNIP2, FAT1, FNBP1, SEC31A, ANXA6, DNM1, DNM2) [61, 77]. Of special interest are ARHGEF11 and CTNND1 splice variants. Both proteins are involved in cell-cell adhesion and the basal B-specific splice variants promote cell migration and invasiveness in several cancer types, such as breast cancer (13,54,74,67). Moreover, depletion of ARHGEF11 in basal breast cancer cells is sufficient to alter cell morphology, which suppresses the cancer cell growth and survival in vitro and in vivo [71]*.* On the other hand, the existence of an isoform-specific antibody for CTNND1 pro-invasive splice variants turns this splicing candidate as a valuable new target to reduce tumour metastasis [78]. ENAH and CD44 are amongst the most studied splicing events impacting cancer and are well-known biomarkers of poor prognosis. ENAH’s inhibition decreases metastasis by slowing down tumour progression and reducing cell invasion and intravasation [79–81]. Whilst the change to basal B splicing signature of CD44, a transmembrane protein that maintains tissue structure, is sufficient to drive an EMT and to increase cell invasion and plasticity by promoting stem cell characteristics [22, 82]. Interestingly, MBNL1 splicing regulation has also been involved in pluripotent stem cell differentiation [83] and cell viability via inhibition of DNA damage response [84]. Promising new splice variants with a potential link with cancer are CSF1, PLOD2, SLK, SPAG9 and TSC2. CSF1 is a macrophage marker which splice variant could correlate with infiltration of tumour-promoting macrophages [73, 85]. Changes in the alternative splicing of the procollagen-lysine PLOD2, which catalyses the deposition and cross-link of collagens in the extracellular matrix, have been intimately linked to EMT progression and cervical, breast, lung, colon and rectal cancer prognosis [40, 86]. Its inhibition reduced proliferation, migration and invasion of cancer cells, while its overexpression promoted cancer stem cell properties and resistance to drugs [62, 87]. SLK was identified as a prognostic biomarker in several cancers and is necessary for the induction of cell migration and invasion during EMT [60, 72, 88]. SPAG9 is a scaffold protein that organises mitogen-activated protein kinases and has been associated with invasion in several types of tumours and prognosis [75, 89, 90]. Finally, TSC2 basal B-specific splicing isoform cannot be phosphorylated by AKT, which leads to a continuously activated mTOR pathway and oncogenic autophagy [74]. More functional studies on the impact of each of these cassette exons splice variants in cancer will increase our knowledge on tumour progression and metastasis with the long term goal of improving diagnostics and treatment. Of note, other types of splicing events, different from the studied cassette exons, have also been shown to play important roles in tumorigenesis, such as alternative splice sites and intron retention [91–93]. It is necessary to extend this type of approaches to all types of splicing events and validate them using independent cohorts of patients. The increase of accessible sequencing data in primary tumours will thus be essential to continue with this type of approaches.

Finally, it is interesting to note that these 25 alternatively spliced exons are basically dependent on three well-known splicing regulators, ESRP1/2 and RBM47, which are intimately linked to EMT and metastasis. ESRP1 is the major regulator of a newly identified epithelial-specific splicing signature [52]. Its expression in cancer cells promotes tumour growth and a mesenchymal-to-epithelial transition which are essential for the formation of new tumours at distal organs during metastasis [94, 95]. RBM47 is a newly identified splicing regulator of EMT that has also been associated with metastasis [56, 96, 97]. Through integrative analysis of clinical breast cancer gene expression datasets, cell line models and mutation data from cancer genome resequencing studies, RBM47 was identified as a suppressor of breast cancer progression and metastasis. It was found mutated in patients with brain metastasis and its expression was necessary to inhibit brain and lung metastatic progression in vivo [56]. Interestingly, despite regulating just 9/25 splicing events of the basal B-specific splicing signature, low expression of RBM47, and not ESRP1, correlated with a poor prognosis and lower survival rate in basal-like breast cancer patients, which increases the interest to design new therapies targeting this splicing regulator.

In fact, this basal B-specific splicing signature has highlighted a subpopulation of basal-like triple negative breast cancer patients differentially expressing several hallmarks of invasive, EMT-like aggressive cancer, such as the newly identified biomarker of metastasis CD36 [20]. CD36 is a fatty receptor expressed in metastasis-initiating cells. Neutralising antibodies that block CD36 completely inhibited the formation of metastasis in orthotopic mouse models of human oral cancer, and CD36 inhibition impaired metastasis in human melanoma and breast cancer-derived tumours. Interestingly, the fatty acid-binding protein 7 (FABP7) correlates with a higher incidence of brain metastasis and lower survival rate in breast cancer patients, which all together points to a potential connection between fatty acid metabolism and metastasis in our subclass of basal-like breast cancer patients [98]. Furthermore, cells expressing our newly identified basal B-specific splicing signature also showed resistance to several EGFR inhibiting drugs. Therapies targeting EGFR have variable and unpredictable responses in breast cancer [99]. By better subclassifying sensitive from resistant tumour cells, diagnoses could be improved, which will impact the choice of treatment and thus the chances of tumour relapse. Extensive drug screening of cells derived from basal B-like patients combined with machine learning strategies to transfer the splicing knowledge obtained will certainly improve the identification of much more suitable treatments for triple-negative breast cancer cells and reduce tumour relapse, thus improving the survival rate.

## Conclusion

Taking advantage of extensive available experimental data in breast cancer cell lines, we performed a knowledge transfer to clinical data to identify the first splicing signature capable of subcategorizing the most aggressive and difficult to treat type of breast cancer, which is basal-like triple negative breast cancer. Based on the pattern of splicing of 25 splicing biomarkers, we could identify two new subclasses of clinically relevant basal-like tumours, basal A and basal B-like, with different sensitivity to drugs and capacity to invade distal organs, which has a direct impact on prognosis. We propose that by testing all basal-like patients with this novel signature, patients with increased chances of creating early metastasis or tumour relapse could be closely monitored to improve their chances of survival. Similarly, by correlating alternative splicing patterns with drug resistance in cancer cell lines, or even cancer cells isolated from patients, more specific splicing biomarkers could be identified for the most adequate and personalised choice of treatment, which is one of the major challenges in triple negative breast cancer. Finally, the newly identified basal B-specific splice variants underline a stem cell-like, highly invasive EMT phenotype, with increased drug resistance, that could be used as novel therapeutic targets to reduce cancer metastasis and relapse, opening new perspectives into the development of improved and more specific treatments for triple negative breast cancer tumours.

## Methods

### RNA-seq transcriptomics analysis: gene expression and alternative splicing

﻿RNA-seq reads were aligned to the human genome (GRCh38, primary assembly) using STAR [100] version 2.5.2b with standard parameters. Gencode v25 (derivated from Ensembl v85) was used for all analysis requiring annotation.

TPMCalculator [101] (v0.0.1) was used to compute transcripts per million (TPM) values and obtain read counts. Q parameter was set to 255 to keep only unique mapped reads and ExonTPM value was used to consider only reads mapped to exons.

Whippet-quant from Whippet software (v10.4) was used to compute Percentage Spliced-In (PSI) values for splicing analysis. Conjointly to Kruskal-Wallis testing, the output from Whippet-quant was further filtered to include only events for which the sum of inclusion counts (IC) and skipping counts (SC) was greater or equal to 10 for both sets of samples. Whippet-delta was used to compute differential splicing (deltaPsi) and probability that there is some change in splicing between conditions. Two heuristic filters were applied on splicing events as advised in whippet documentation; |deltaPsi| > 0.1 and P(|deltaPsi| > 0.0) > = 95% were considered reliable parameters to filter biologically relevant AS events.

When necessary, Biobambam2 [102] (v 2.0.87) was used to transform bam files into fastq in order to be processed by Whippet.

Gene ontology (GO) analysis was done using the DAVID (v 6.8) [103] functional annotation tool (https://david.ncifcrf.gov/home.jsp) using Benjamini-Hochberg adjusted *P* value cutoff of 0.05 to define a term as enriched. Go terms enrichment was restricted to GOTERM BP-FAT, GOTERM MF-FAT, and GOTERM CC-FAT, KEGG_PATHWAY and REACTOME_PATHWAY.

Gene Set Enrichment Analysis (GSEA v20.0.5) was carried out on the GenePattern [104] web platform using phenotype for permutation type and 1000 for the number of permutations to execute. FDR cutoff of 25% for potential true positive finding was used as documented in the GSEA user guide. Read counts were previously normalised using DESeq2 [105] (v 1.10.1) on the same Platform.

R version 3.6.2 was used all along this study excepted for GSEA.

All heatmaps were done online using Morpheus https://software.broadinstitute.org/morpheus/. Values were adjusted by *Z*-score. (subtract mean and divide by standard deviation). Hierarchical clustering was done in Morpheus. We selected “Metric One minus Pearson correlation” as a measure of distance between pairs of observation and “Average” as the linkage method. The clusters were done using rows and columns together. Columns were grouped by cancer subtypes.

Sashimi plots to look cassette exons events were done using ggsashimi tool [106].

### Machine learning and feature selection

First, we construct a classifier to distinguish basal B/A cell lines using a Random Forest with 1000 trees. After, we applied this model to the TCGA patients. Based on Gini impurity, we computed the class probability to predict patient labelled as B-like or A-like. Then, mixing initial cell lines with a subset of patients classified with the more reliability (the ones picked up with higher class probability not passing below a threshold of *P* = 0.6), we create a new model. Each addition of patients is called a round, during which a new model is created, giving new predictions (probabilities) for the remaining patients. By limiting the number of new patients added at each round (10 x n_current_round) (Fig. 3c and Additional file 1: Figs. S3c-4c), the model can gradually learn from the patient data and avoid overfitting. With such conditions, we can observe a gradual shifting in feature importance from the ones informative to classify cell lines to the ones informative to classify patients and cell lines (Fig. 3d and Additional file 1: Figs. S3d-4d). The algorithm stops when it can no longer incorporate the patients into one or the other group given the cutoff of *P* = 0.6. ML analyse was done with Python 3.7.3 based on scikit-learn version 0.21.2.

To select the more efficient features that were able to separate B-like from A-like patients, we used Boruta package (0.3) implemented in python. We ran it 10 times with different random states, on the 217 features related to splicing and kept the ones that were present at least 7 times on 10. We ended with 25 AS features. Considering only these 25 AS features, we applied TSNE function from manifold package (with perplexity = 20) to 3 other datasets of basal cell lines (*n* = 56) to check the features were sufficient to distinguish spatially these cell lines according to their labels.

For the classification using only differentially expressed genes (Additional file 1: Fig. S3) or a mix of differentially spliced and expressed features (Additional file 1: Fig. S4), we applied the same strategy using the information from the 635 differentially expressed genes and the 217 differentially spliced exons scaling independently the values from the cell lines and patients with sklearn’s StandardScaler. We also had to reduce the probability threshold to 0.55 in the mixed model.

### Breast cancer annotation

Basal B and A cells were labelled according to literature: Neve et al. [28], Kao et al. [33], Marcotte et al. [107], Dai et al. [108]. PAM50 intrinsic subtype was retrieved from https://www.cell.com/cancer-cell/fulltext/S1535-6108(18)30119-3 [109].

Claudin Low status was defined with script downloaded from https://github.com/clfougner/ClaudinLow/blob/master/Code/TCGA.r [110] using dataset from http://download.cbioportal.org/brca_tcga_pan_can_atlas_2018.tar.gz [111, 112].

### Survival analysis

Log-rank tests were performed using the functions surv and survfit from R package (survival v3.1.8). A different survival was considered significative if log rank test *p* value was < 0.05. Coxph function was also used for univariate Cox regression analysis in order to compute Hazard Ratio and 95% Interval of confidence. Kaplan-Meier curve was plotted using function ggsurvplot from R package survminer (0.4.6). Plots were truncated at 5 years, but the analyses were conducted using all of the data. All endpoints used for survival analysis in this study were retrieved from this study [113].

### Statistics

Wilcoxon rank-sum test was used to assess statistical significance within boxplots.

They were noted. *P* < 0.05 (∗), *P* < 0.01 (∗∗), and *P* < 0.001 (∗∗∗), *P* < 0.0001 (∗∗∗∗).

Kruskal-Wallis test was used to keep differential features for expression (TPM values) or splicing (PSI values) when Luminal, basal A and B cell lines were compared and displayed in heatmap figures. A threshold of *p* value < 10–5 was used to filter out potential false positive and reduce the number of features in order to apply hierarchical clustering. This threshold was adapted depending on the number of samples in the comparison. For RNA binding proteins, a higher cut off of *p* < 10–9 was used because 5 projects were pulled together.

## Supplementary Information

**Additional file 1: Fig. S1.** Allele-specific alternative splicing and its functional genetic variants in human tissues. **Fig. S2.** Hierarchical clustering and k-means of patients based on differential gene expression and splicing. **Fig. S3.** Semi-supervised Random Forest Classifier to transfer cell lines knowledge to patients using expression levels. **Fig. S4.** Semi-supervised Random Forest Classifier to transfer cell lines knowledge to patients using alternative splicing and expression levels. **Fig. S5.** In silico validation of basal B splicing signature. **Fig. S6.** Prognostic value of individual alternatively spliced genes from the basal B-specific signature.

**Additional file 2: Table S1.** GEO accession numbers for all the datasets analysed.

**Additional file 3: Table S2.** Name, coordinates (Hg38) and PSI mean value and standard error for the 25 exons of the basal B-specific signature in Basal A and Basal B cancer cells and patients. The difference in splicing levels between basal B and basal A is shown as deltaPSI.

## Data Availability

All datasets are available in the Gene Expression Omnibus (GEO): GSE75489, GSE58381, GSE75491, GSE61220, PRJEB25042, GSE74881, GSE75492, PRJNA523380, PRJNA297219, PRJNA210428, PRJNA251383, PRJEB30617 (detailed in Additional file 2: Table S1) and The Cancer Genome Atlas (TCGA) repositories upon request (https://www.ncbi.nlm.nih.gov/projects/gap/cgi-bin/study.cgi?study_id=phs000178.v11.p8).

## Abbreviations

AS: Alternative splicing
CE: Cassette exons
EMT: Epithelial-to-mesenchymal transition
CSC: Cancer stem cells
CTC: Circulating tumour cells
PSI: Percentage spliced-in
TPM: Transcripts per million
DSS: Disease-specific survival
TCGA: The Cancer Genome Atlas
RBPs: RNA binding proteins
