# Supplementary material for: A cell-to-patient machine learning transfer approach uncovers novel basal-like breast cancer prognostic markers amongst alternative splice variants
Source: BMC Biol. 2021 Apr 12;19:70. doi: 10.1186/s12915-021-01002-7 (PMC8042689; doi:10.1186/s12915-021-01002-7)
Supplement: Supplementary file 1 — Additional file 1: Fig. S1. Allele-specific alternative splicing and its functional genetic variants in human tissues. Fig. S2. Hierarchical clustering and k-means of patients based on differential gene expression and splicing. Fig. S3. Semi-supervised Random Forest Classifier to transfer cell lines knowledge to patients using expression levels. Fig. S4. Semi-supervised Random Forest Classifier to transfer cell lines knowledge to patients using alternative splicing and expression levels. Fig. S5. In silico validation of basal B splicing signature. Fig. S6. Prognostic value of individual alternatively spliced genes from the basal B-specific signature. [file 12915_2021_1002_MOESM1_ESM.pdf]

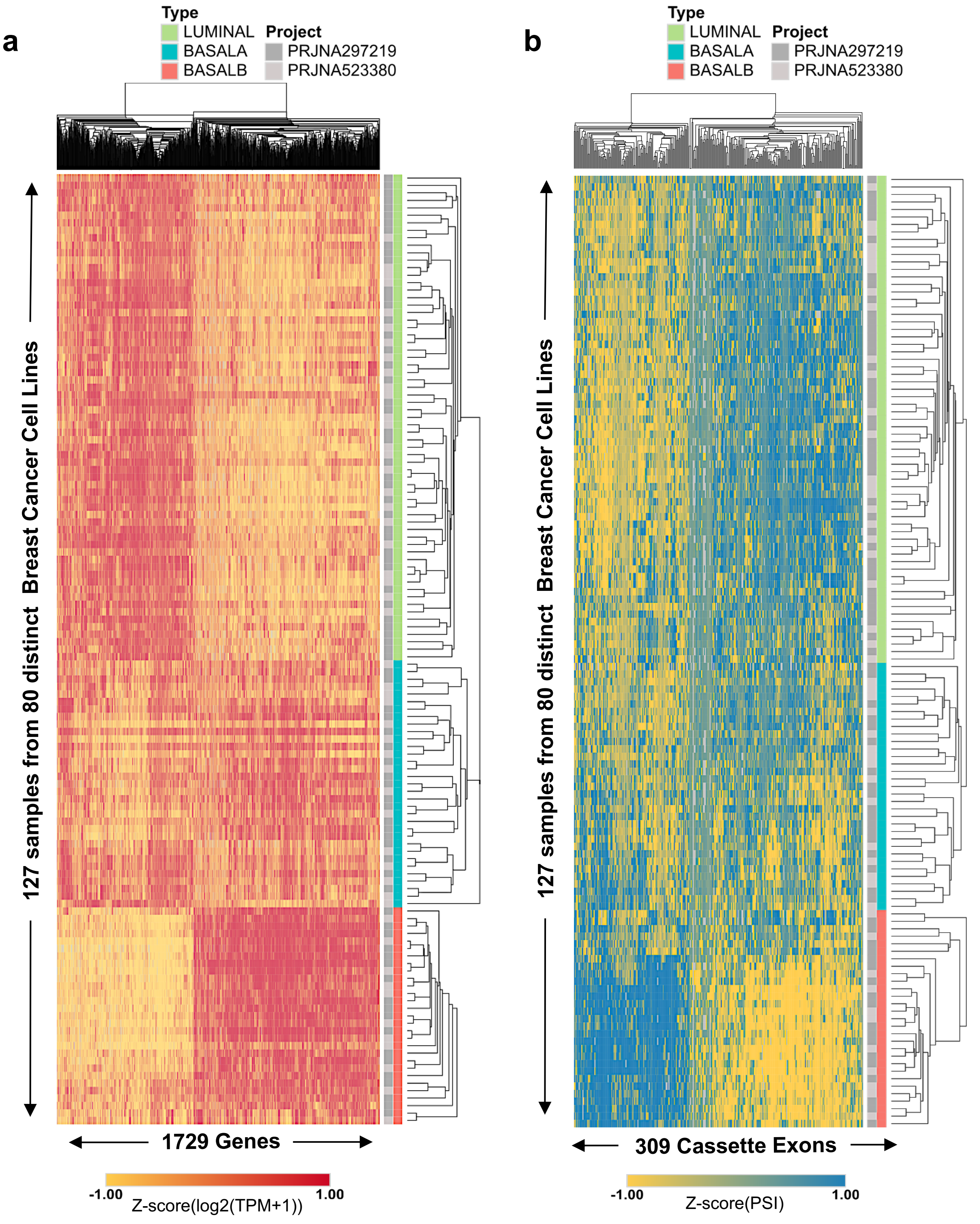
a
Type
LUMINAL
BASALA
BASALB
Project
PRJNA297219
PRJNA523380
127 samples from 80 distinct Breast Cancer Cell Lines
1729 Genes
-1.00
1.00
Z-score(log2(TPM+1))
b
Type
LUMINAL
BASALA
BASALB
Project
PRJNA297219
PRJNA523380
127 samples from 80 distinct Breast Cancer Cell Lines
309 Cassette Exons
-1.00
1.00
Z-score(PSI)

Figure S1

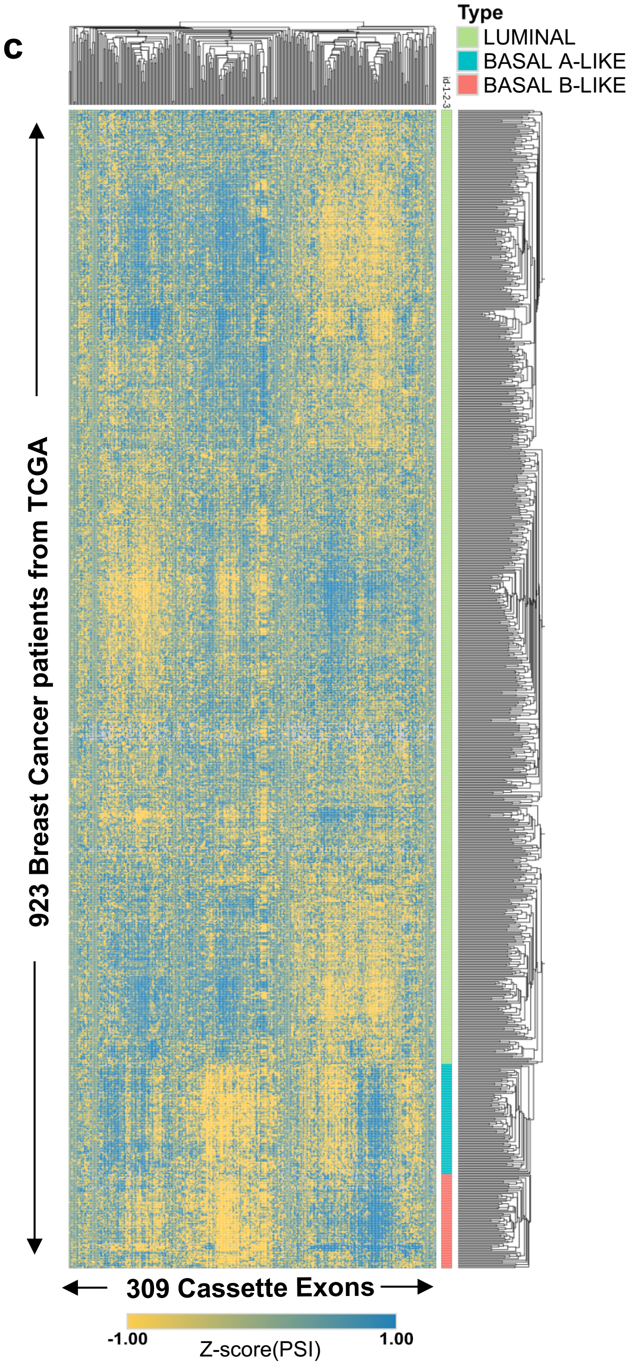

Figure S1. Differential clustering of basal B cell lines based on gene expression and splicing patterns. **a.** Heatmap of gene expression levels, in Transcripts per Million (TPM) values, of 1729 genes differentially regulated between Luminal, basal A and basal B cell lines (P-value < 10^{-5} by Kruskal-Wallis Test). **b.** Heatmap of exon inclusion levels, using Percentage Spliced-In (PSI), of 309 exons differentially spliced between luminal, basal A and basal B cell lines (P-value <10^{-5} by Kruskal-Wallis Test). **c.** Heatmap of exon inclusion levels, using Percentage Spliced-In (PSI), of the 309 exons differentially spliced between cell lines in the 923 luminal and basal-like breast cancer patients available from the TCGA. We separate the basal-like patients in basal A-like and basal B-like based on the signature found in the cell lines.

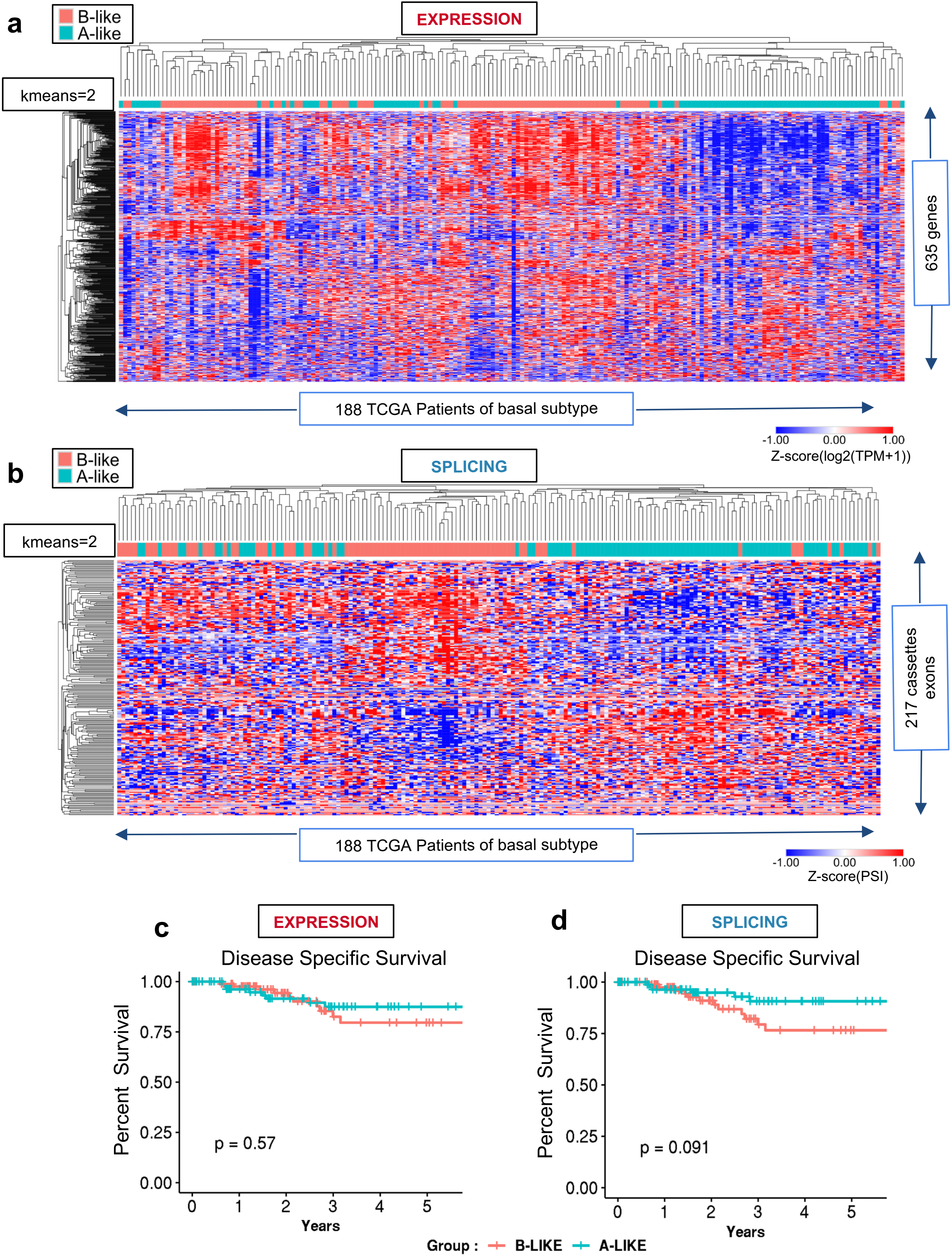
a
B-like
A-like
EXPRESSION
kmeans=2
635 genes
188 TCGA Patients of basal subtype
-1.00 0.00 1.00
Z-score(log2(TPM+1))
b
B-like
A-like
SPLICING
kmeans=2
217 cassettes exons
188 TCGA Patients of basal subtype
-1.00 0.00 1.00
Z-score(PSI)
c
EXPRESSION
Disease Specific Survival
Percent Survival
p = 0.57
Years
d
SPLICING
Disease Specific Survival
Percent Survival
p = 0.091
Years
Group : B-LIKE A-LIKE

Figure S2

Figure S2. Hierarchical clustering and k-means of patients based on differential gene expression and splicing. a-b. Using 188 TCGA patients classified as basal-like breast cancer, we applied hierarchical clustering followed by a k-means (n=2**)** on expression (**a**) or splicing values (**b**) characteristic of basal B cell lines. Each time, K-means distinguished two groups we named “B-like” (red) and “A-like” (blue). In **a,** k-means was applied to TPM expression values for the 635 genes differentially expressed between basal A and B cell lines, which were displayed in the heatmap annotated Expression. In **b,** k-means was applied to PSI values of the 217 differentially spliced exons between basal A and basal B cell lines, which were displayed in the heatmap annotated Splicing. **c,d.** Kaplan-Meier plots of disease specific survival (DSS) of basal-like breast cancer patients previously separated in two groups by the k-means algorithm (k=2) for expression and splicing. Logrank test p-values (P) between “B-like” (red line) and “A-like” (blue line) patient groups are shown.

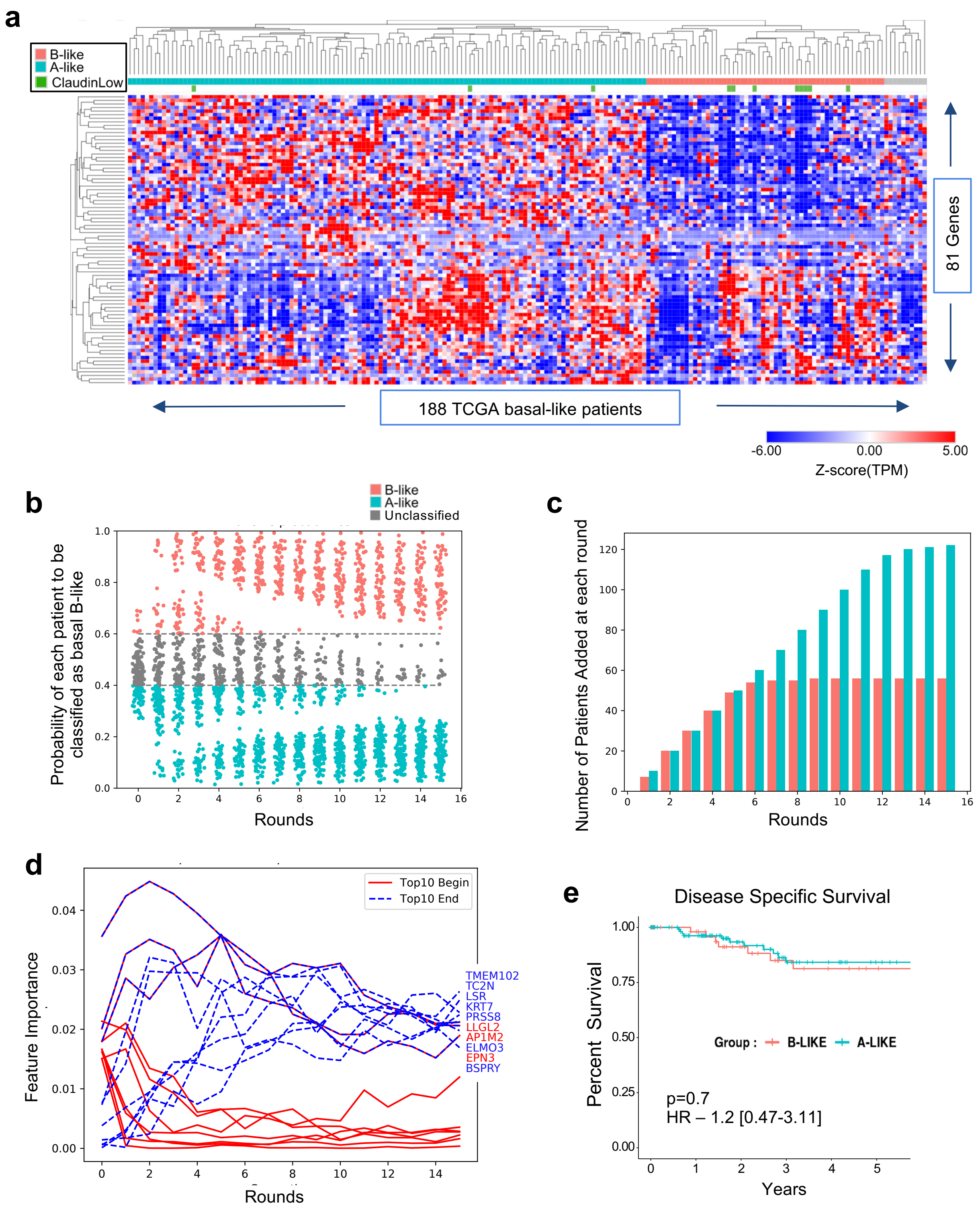
a
B-like
A-like
ClaudinLow
81 Genes
188 TCGA basal-like patients
-6.00
0.00
5.00
Z-score(TPM)
b
B-like
A-like
Unclassified
Probability of each patient to be classified as basal B-like
Rounds
c
Number of Patients Added at each round
Rounds
d
Top10 Begin
Top10 End
Feature Importance
Rounds
TMEM102
TC2N
LSR
KRT7
PRSS8
LLGL2
AP1M2
ELMO3
EPN3
BSPRY
e
Disease Specific Survival
Percent Survival
Group : B-LIKE A-LIKE
p=0.7
HR – 1.2 [0.47-3.11]
Years

Figure S3

Figure S3. Semi-supervised Random Forest Classifier to transfer cell lines knowledge to patients using expression levels. a. Heatmap of 81 genes TPM values for TCGA basal-like patients predicted as basal B-like (red) or basal A-like (blue) by the semi-supervised random forest classifier based on gene expression levels. Claudin low tumors are highlighted in green. Only the best features are represented. **b.** For all patients, we plot their probabilities to be classified as basal B-like, basal A-like or unclassified at each round. Dotted lines indicate thresholds used to classify a patient as basal B-like (>0.6) or basal A-like (<0.4)**. c.** Bar plot showing the number of patients added at each round. Patients with the highest probability to be classified are sequentially incorporated to the input cell lines in order to create a new classifier for the next round of integration. **d**. Evolution of the feature importance at each round of iterative training. In red are the 10 splicing variants (features) most informative at the beginning of the transfer learning process. In blue are the 10 splicing variants most informative at the end. Only three exons remained informative from the beginning to the end (in blue and red). The name of the top 10 final most informative spliced genes are presented in sequential order. **e.** Kaplan-Meier plots of disease specific survival in patients classified as basal A-like (blue) and basal B-like (red) based on gene expression patterns. Hazard ratio (HR) and logrank p-value (P) discriminating the two groups are shown.

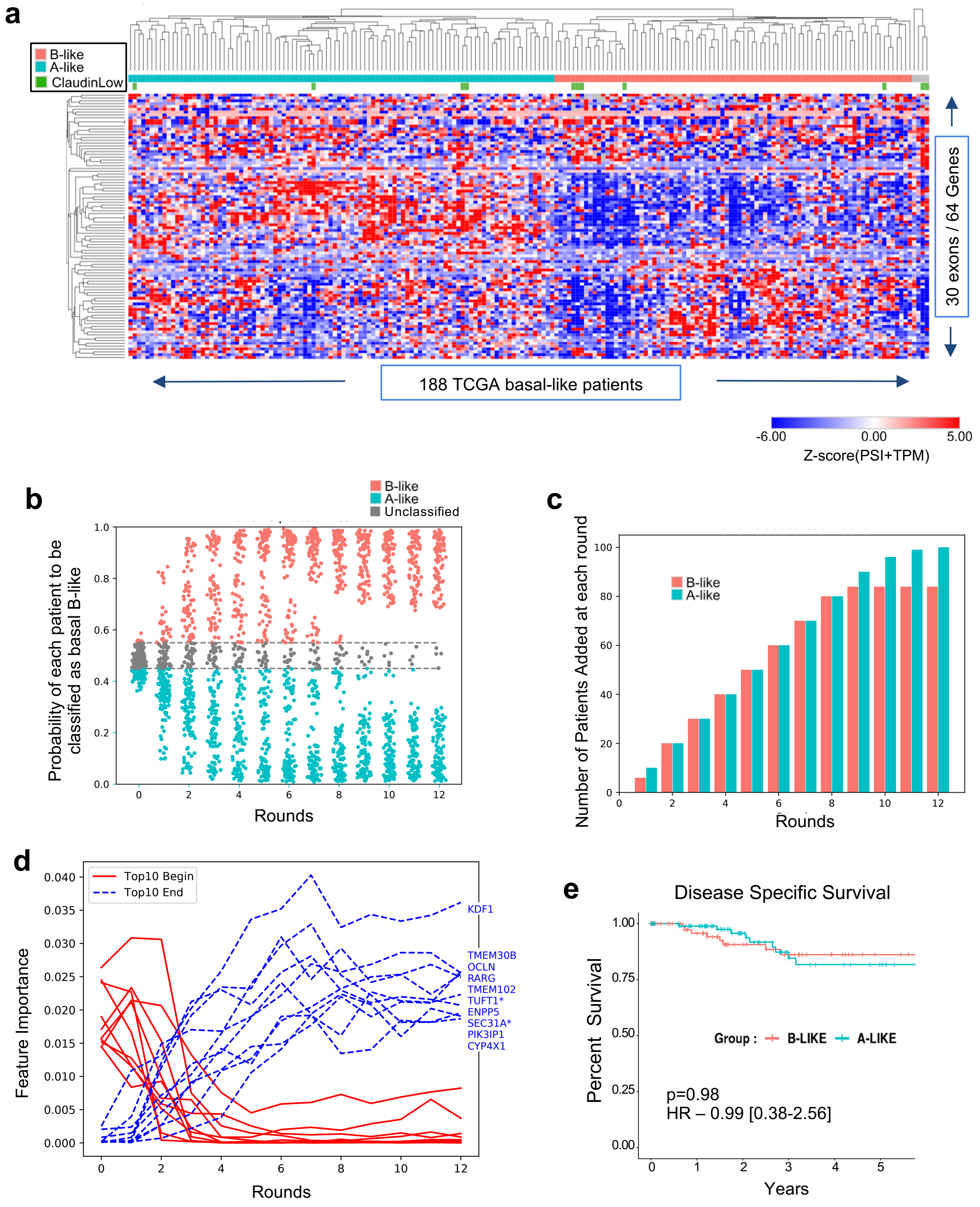
a
B-like
A-like
ClaudinLow
30 exons / 64 Genes
188 TCGA basal-like patients
-6.00
0.00
5.00
Z-score(PSI+TPM)
b
B-like
A-like
Unclassified
Probability of each patient to be classified as basal B-like
Rounds
c
Number of Patients Added at each round
B-like
A-like
Rounds
d
Top10 Begin
Top10 End
Feature Importance
Rounds
KDF1
TMEM30B
OCLN
RARG
TMEM102
TUFT1*
ENPP5
SEC31A*
PIK3IP1
CYP4X1
e
Disease Specific Survival
Percent Survival
Group : B-LIKE A-LIKE
p=0.98
HR – 0.99 [0.38-2.56]
Years

Figure S4

Figure S4. Semi-supervised Random Forest Classifier to transfer cell lines knowledge to patients using splicing and expression levels. a. Heatmap of 30 exons PSI values and 64 genes TPM values for TCGA basal-like patients predicted as basal B-like (red) or basal A-like (blue) by the semi-supervised random forest classifier based on differential splicing and gene expression levels. Claudin low tumors are highlighted in green. Only the best features are represented. **b.** For all patients, we plot their probabilities to be classified as basal B-like, basal A-like or unclassified at each round. Dotted lines indicate thresholds used to classify a patient as basal B-like (>0.55) or basal A-like (<0.4)**. c.** Bar plot showing the number of patients added at each round. Patients with the highest probability to be classified are sequentially incorporated to the initial model in order to create a new classifier for the next round of integration. **d**. Evolution of the feature importance at each round of iterative training. In red are the 10 splicing variants (features) most informative at the beginning of the transfer learning process. In blue are the 10 splicing variants most informative at the end. The name of the top 10 final most informative spliced genes are presented in sequential order. With an asterisk we indicate the features that correspond to splicing events **e.** Kaplan-Meier plots of disease specific survival in patients classified as basal A-like (blue) and basal B-like (red) based on gene expression patterns. Hazard ratio (HR) and logrank p-value (P) discriminating the two groups are shown.

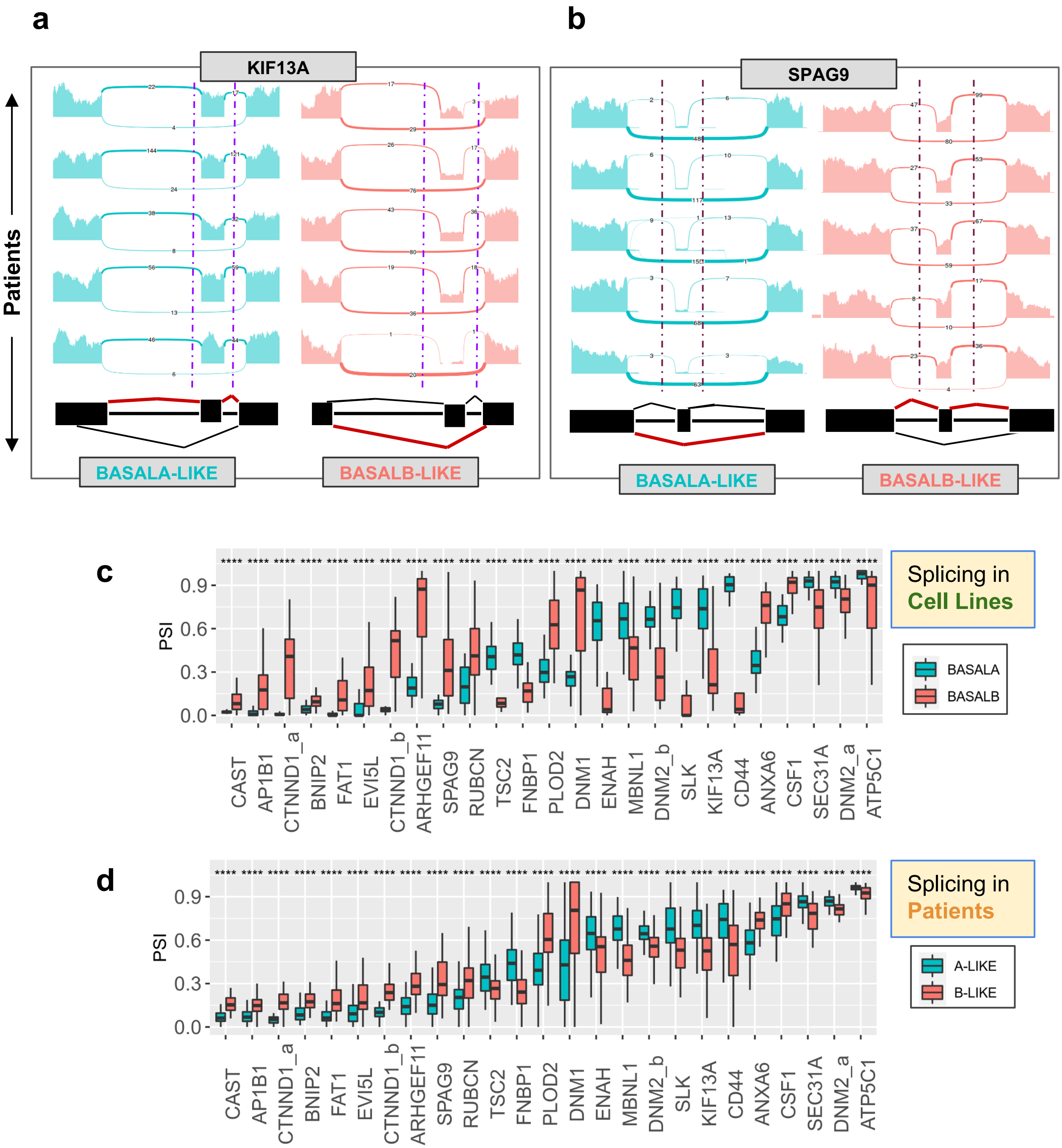

Figure S5. ***In silico*** **validation of basal B splicing signature. a,b.** Sashimi plots of KIF13A and SPAG9 patterns of splicing in randomly selected basal A-like and basal B-like patients. **c,d.** Box plots of the median and 25th percentile of the Percent Spliced-In (PSI) values for the 25 cassette exons in basal A/B cell lines and basal A-like/B-like patients. **** P <0.0001 in Wilcoxon rank-sum test comparing A to B.

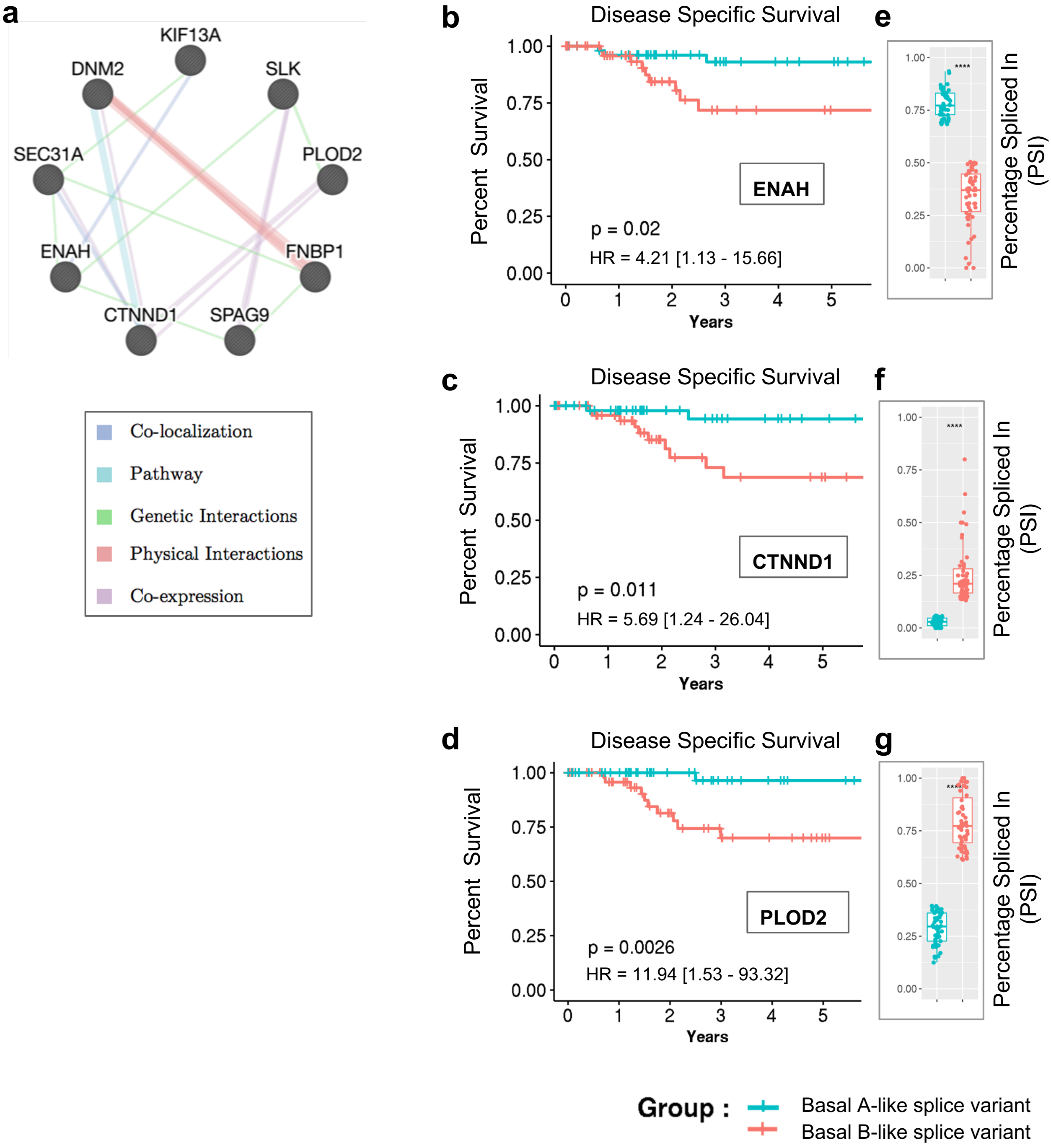

Figure S6. Prognostic value of individual alternatively spliced genes from the basal B-specific signature. a. Network of functional association (GeneMania) between RBM47-dependent spliced genes from the 25 basal B-specific splicing signature. **b,c,d.** Kaplan-Meier curves of disease specific survival in patients expressing basal A-like (blue) or basal B-like (red) ENAH, CTNND1 and PLOD2 splice variants grouped by PSI terciles. Hazard ratio (HR) and respective logrank p-values (P) discriminating groups are shown. **e,f,g.** Box plots of the median and 25th percentile of the PSI values of the patients used in the survival curves.
